# Insights into Advances and Applications of Biomaterials for Nerve Tissue Injuries and Neurodegenerative Disorders

**DOI:** 10.1002/mabi.202400150

**Published:** 2024-09-30

**Authors:** Varsha Pai, Bhisham Narayan Singh, Abhishek Kumar Singh

**Affiliations:** ^1^ Manipal Centre for Biotherapeutics Research Manipal Academy of Higher Education Manipal Karnataka 576 104 India; ^2^ Department of Biotechnology Manipal School of Life Sciences Manipal Academy of Higher Education Manipal Karnataka 576 104 India

**Keywords:** nanoparticles, nerve tissue injury, neurodegeneration, stem cells, tissue engineering

## Abstract

The incidence of nerve tissue injuries, such as peripheral nerve injury, spinal cord injury, traumatic brain injury, and various neurodegenerative diseases (NDs), is continuously increasing because of stress, physical and chemical trauma, and the aging population worldwide. Restoration of the damaged nervous system is challenging because of its structural and functional complexity and limited regenerative ability. Additionally, there is no cure available for NDs except for medications that provide symptomatic relief. Stem cells offer an alternative approach for promoting damage repair, but their efficacy is limited by a compromised survival rate and neurogenesis process. To address these challenges, neural tissue engineering has emerged as a promising strategy in which stem cells are seeded or encapsulated within a suitable biomaterial construct, increasing cell survival and neurogenesis. Numerous biomaterials are utilized to create different types of constructs for this purpose. Researchers are trying to develop ideal scaffolds that combine biomaterials, cells, and molecules that exactly mimic the biological and mechanical properties of the tissue to achieve functional recovery associated with neurological dysfunction. This review focuses on exploring the development and applications of different biomaterials for their potential use in the diagnosis, therapy, nerve tissue regeneration, and treatment of neurological disorders.

## Graphical abstract

This review addresses different types of biomaterials and their applications in treating nerve tissue injuries and neurodegenerative disorders. Moreover, it highlights several FDA‐approved biomaterial‐based products and ongoing clinical trials on biomaterials for nerve tissue regeneration.

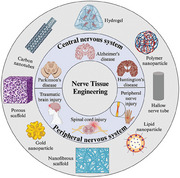

## 1 Introduction

**Figure 1:**
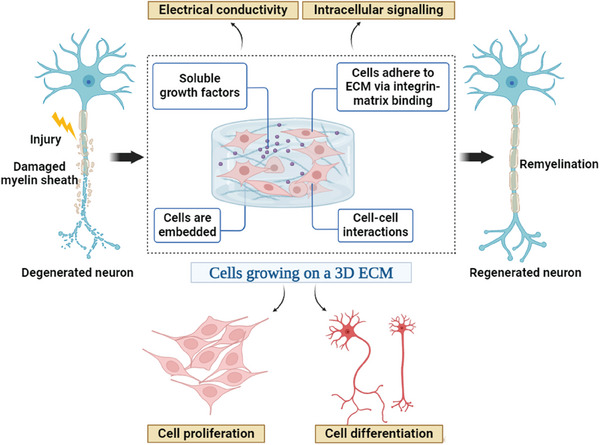
Biomaterials are combined with growth factors and cells that mimic the extracellular matrix (ECM) of damaged nerve tissues and are used in nerve regeneration. The application of biomaterials in the regeneration of damaged nerves will increase the body's natural healing process, support the proliferation and survival of nerve cells, and lead to better functional recovery from nerve injuries.

Neurological diseases are caused by the progressive deterioration of neurons in both the spinal cord and different parts of the brain, leading to neurodegenerative disorders (NDs),^[^ ^1^ ^]^ dyskinesia, behavioral alterations, and intellectual aberrations.^[^ ^2^ ^]^ Most NDs can be sporadic or familial, depending on the family medical history.^[^ ^3^ ^]^ Although most NDs do not affect elderly individuals, they can still be applied to normal brain development among young individuals. Regenerative medicine holds great promise for addressing neurological diseases. With advances in regenerative medicine, autografts and allografts have been used and are considered the gold standard for regenerating damaged nerves.^[^ ^4^ ^]^ The disadvantages of the autologous graft include a shortage of supplies, a secondary incision for removing graft tissues, incompatibility of fascicles, permanent loss of nerve functions, and scarring at the implanted site.^[^ ^5^ ^]^ However, allografts require systemic immunosuppression, and an increase in morbidity associated with immunomodulatory therapy poses a major concern.^[^ ^6^ ^]^ Many artificial and biological nerve grafts have been reported to replace traditional auto/allo/xenografts.^[^ ^7^ ^]^ Like other engineered tissues, neural tissue‐engineered grafts also incorporate cells and growth factors in fabricated scaffolds to replace traditional grafts.^[^ ^8^ ^]^ The rise of regenerative medicine can be linked to a more specialized field of neural tissue engineering.^[^ ^7^ ^]^ Restoring impaired nerve function and promoting the regeneration of damaged neurons is possible with stem cell transplantation (**Figure** **1**).^[^ ^9^ ^]^

Stem cell therapy mitigates some of the limitations of conventional therapies while simultaneously promoting the innate ability of injured tissue to regenerate itself through the release of growth factors and cytokines, as well as regulating the immunomodulatory response after injury.^[^ ^10^, ^11^ ^]^ The supply of stem cells should be easily available and allow for quick and regulated neurogenesis. Under certain circumstances, mesenchymal stem cells (MSCs) can differentiate into Schwann cells in response to certain stimuli and thus stimulate the regeneration of damaged nerves.^[^ ^12^ ^]^ The proangiogenic potential of MSCs is an essential mechanism for the regeneration of damaged tissues, and stem cell therapies, in general, have shown good potential in treating spinal cord injury (SCI). Vascular endothelial cells, when cotransplanted with MSCs, improved angiogenesis, but there were issues with the immunogenicity of these cells. The use of exosomes derived from vascular endothelial cells to activate MSCs offers a viable approach to effectively treat SCI by leveraging the role of exosomes in intracellular interactions and vascular remodeling.^[^ ^13^ ^]^ Conditioned media of bone‐derived MSCs can help to cure subacute and acute peripheral injuries in rabbits, with an efficacy similar to that of bone marrow‐derived MSCs in healing damaged nerves.^[^ ^9^ ^]^ However, the use of stem cells alone has various drawbacks, such as limited availability and high cost.

**Figure 2:**
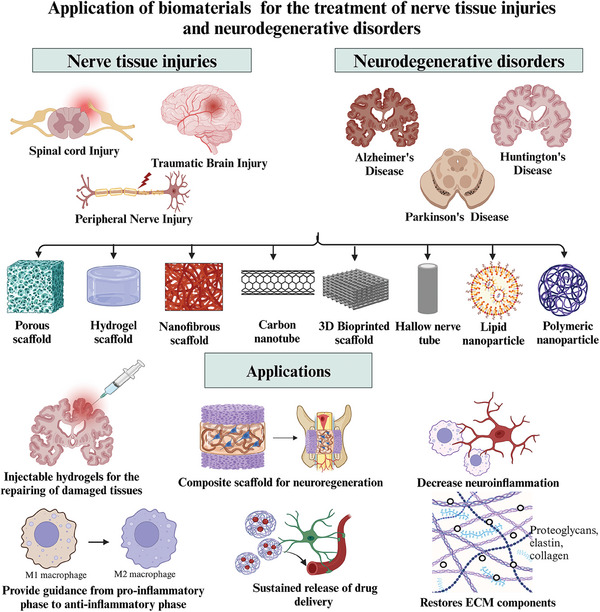
Overview of the applications of biomaterials in treating nerve tissue injuries and neurodegenerative disorders, including the use of polymeric, composite, and nanobiomaterials engineered to facilitate nerve regeneration, improve cell adhesion, and deliver therapeutic agents, thereby supporting the recovery and treatment of damaged neural tissues and addressing neurodegenerative conditions.

A combination of stem cells and biomaterials has the potential to enhance the recovery of injured nerve tissues. Tissue‐engineered nerve grafts have shown potential in the treatment of various nerve tissue injuries and NDs, as highlighted in **Figure** **2**.

**Figure 3:**
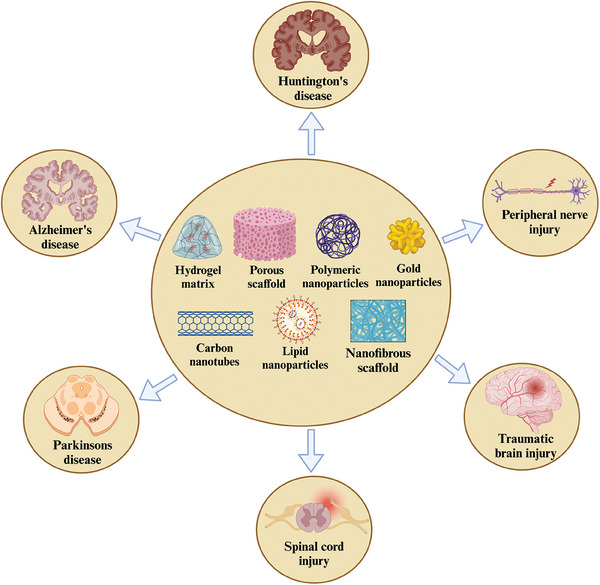
Various biomaterials have been employed for the treatment of neurological disorders. These materials include hydrogels, fibrous scaffolds, and polymers that are used to design biomaterials to support nerve tissue repair and regeneration. These biomaterials are designed to release growth factors, mimic the natural ECM, and provide support to damaged neurons. They serve to increase cellular growth, promote functional recovery, and improve integration with surrounding tissue.

The human nervous system is one of the most intricate networks and requires a much more sophisticated 3D scaffold and structural intricacy.^[^ ^14^ ^]^ The main principle of tissue engineering involves emulating the microenvironment of native tissues, which includes characteristics such as mechanical properties, electrical signals, structural organization, and chemical composition.^[^ ^15^ ^]^ Different types of scaffolds, such as hydrogels, nanofibers, porous scaffolds, carbon nanotubes, and polymeric, gold, and lipid‐based nanoparticles, have been fabricated and specifically tailored to encourage the growth and repair of damaged neurons (**Figure** **3**). This approach holds potential solutions for conditions with limited treatment options, such as spinal cord injuries. Although the FDA has approved several nerve‐repairing devices, the persistence of challenges in treatment and recovery promotes the necessity for novel tissue engineering methods in therapeutics and research.^[^ ^15^ ^]^ In this review, we discuss preclinical development and current clinical advancements in neurological disorders, with an emphasis on the potential of 3D biomaterials in neurogenesis and neurological dysfunction.

## 2 Biomaterials for Improving Neurogenesis

### 2.1 Characteristics of Biomaterials

Biomaterials are tissue‐compatible substances with minimal to no harmful reactions upon implantation. Biomaterials have been developed to be incorporated into biological tissues, mainly for diagnostic or medical purposes.^[^ ^16^ ^]^ The tissue engineering applications of biomaterials, both synthetic and natural, have been widely recognized because of their distinctive biocompatibility, nonimmunogenic characteristics, and significant regeneration potential.^[^ ^16^, ^17^ ^]^ In the regeneration of tissues, biomaterials with various compositions have demonstrated tremendous potential. For example, scaffolds with chemical and surface modifications are highly effective at controlling the adhesion, migration, proliferation, and differentiation of incorporated cells, which in turn controls the regeneration of damaged tissues in vivo and in vitro.^[^ ^18^ ^]^ These biomaterials are necessary for administering stem cells, bridging the lesion site, mitigating inhibitory neurite factors, and releasing bioactive molecules such as drugs, growth factors, and antibodies.

Synthetic biomaterials facilitate control over critical parameters such as stiffness, porosity, degradability, and microstructure. Nonetheless, the biocompatibility of these synthetic biomaterials is impacted by the absence of recognition sites.^[^ ^19^ ^]^ The drawbacks of synthetic biomaterials include the absence of natural cell adhesion sites that need chemical alterations to improve cell attachment.^[^ ^20^ ^]^ By using advanced approaches and various synthetic routes, it is possible to modify existing polymers and alter their characteristics for use in various biomedical applications, such as drug delivery and repair of damaged tissues. Because of their metabolic compatibility, biological systems can easily absorb natural polymers such as gelatin, collagen, elastin, and others. However, natural biomaterials have several disadvantages, such as decreased mechanical strength, the possibility of immunogenicity, and structural complexity.^[^ ^21^ ^]^

Some natural polymers, such as collagen, are essential to the ECM and are biodegradable, biocompatible, and less toxic. It supports cell adhesion and proliferation. However, they degrade very quickly under in vivo conditions. Chitosan, derived from chitin, is biodegradable and biocompatible and promotes the regeneration of nerves. However, these natural polymers lack mechanical properties. These natural polymers are combined with synthetic polymers such as polylactic coglycolic acid (PLGA), which is a copolymer of lactic acid and glycolic acid. It is biodegradable, with degradation rates when combined with different polymer compositions.^[^ ^22^ ^]^ Polycaprolactone (PCL) is a synthetic polymer made from caprolactone monomers. It is semicrystalline in nature. However, because of their slow degradation rate and good mechanical strength, they have been widely used in implants.

By combining both types of polymers, composite biomaterials can be synthesized to overcome the disadvantages of natural and synthetic polymers. Appropriate degradability and biocompatibility can be achieved by combining both natural and synthetic polymers either chemically or physically.^[^ ^21^ ^]^ Biocompatible polymers such as gelatin, silk fibrin, chitosan, and poly(n‐butyl cyanoacrylate) have been used to treat a variety of diseases, as they cause minimal foreign body reactions. Thus, for the regeneration and repair of damaged nerves, biomaterials with the ability to regulate cell behavior and the tissue microenvironment are essential.^[^ ^22^ ^]^

Current problems in cellular models of brain development might be addressed by biomaterials. Biomaterials are defined broadly as any substance created to interact with individual cells or cell constructions. These materials have distinctly different chemical compositions and structural characteristics and are produced via various synthetic techniques. A variety of biomaterials that cause morphological and physiological changes in cells are produced by deliberately modifying these characteristics.^[^ ^23^ ^]^ Different types of bioactive molecules have been used in combination with biomaterials to enhance neurogenesis, as highlighted in **Table** **1**.

**Table 1:** Types of biomaterials, bioactive molecules and their structure and properties used in preclinical studies for various neurological dysfunction and are involved in neurogenesis. Structure of molecules were taken from PubChem.

Biomaterial	Neuro bioactive molecules, structure & properties	Scaffold	Neurological dysfunction	Preclinical/clinical outcomes	Refs.
Collagen	IL‐4 molecules loaded in ZIF‐8NPs. **Properties**: Protection of nerve tissue from inflammatory damage. Thus, enhancing the repair of CNS repair. Involved in the polarization of IL‐4. Provides neuroprotective effect Bone marrow mesenchymal stem cell‐derived exosomes (BME) incorporated into Hyaluronan‐collagen based hydrogel. **Properties**: MSCs have the potential for cell‐free therapy of various disorders. Promote angiogenesis and enhance regeneration of damaged tissues. Induce axonal regeneration, by regulation of immunoreaction. Decrease in inflammation.	Hyaluronan‐collagen based hydrogel	Spinal cord injury (SCI) Traumatic brain injury	**In vitro**: BME incorporated into hydrogel was taken up by the neural stem cells and HUVECs cells. Promoted differentiation of neural stem cells into oligodendrocyte and mature neurons. Inhibiting their differentiation into astrocytes. Involved in enhancing angiogenesis at the lesion site. Axonal regeneration, remyelination, synaptic formation, and even brain structural remodeling was achieved. **In Vivo**: In SD rats, these microfibers enhanced the infiltration of M2 macrophages after polarization. Significant improvements in neurological function. Suppress the inflammatory responses and inhibit the formation of glial scars.	[140, 141]
PCL	**Melatonin Structure**: 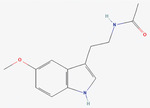 **Properties**: Anti‐inflammatory, Scavenging of free radical, antioxidant properties. Shown a neuroprotective effect and helps in decreasing scar **Citicoline Structure**: 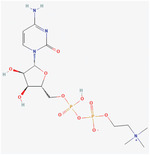 **Properties**: Component of neuron membrane. Preclinical studies have shown that citicoline is beneficial for PD, cognitive impairment, and head trauma. It has shown neuroprotective effects and topical administration, and IP has increased the regeneration and recovery of function of PNI.	PCL‐gelatin‐based fibrous	Peripheral nerve injury (PNI)	**In Vitro**: Improved differentiation and proliferation of PC12 cells. Enhances neurite formation with increased concentration of melatonin. Increase expression of MAP2. Facilitate regeneration of damaged peripheral nerve. **In vivo**: Induced neuroprotective effects through regeneration in Wistar rats. Increased growth of nerve cells, regeneration of damaged nerves, and provided functional recovery.	[142, 143, 144]
Alginate	**Citric acid functionalized graphite nanofilaments** 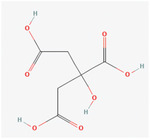 **Properties**: Improve alginate hydrogel's electrical, mechanical, and biological properties for neural tissue engineering. Citric acid functionalization is a simple approach to induce the formation of oxygen‐containing functional groups on the surface of graphite nanofilaments, thereby assuring their uniform distribution within the alginate matrix. Provide local conductive zones, which enhance intracellular signaling of neural cells.	Citric acid functionalized graphite nanofilaments ‐Alginate hydrogels	PNI	**In vitro**: Improved differentiation and proliferation of PC12 cells. Enhances neurite formation. **In vivo**: No inflammatory response after implantation in the Guinea pig. No giant cell reaction and fibrosis.	[145]
pDNM	**Incorporation of Growth factor (NGF and VEGF)** **Properties**: Induce neuron regeneration and vascularization. VEGF will stimulate the formation of blood vessels. NGF is involved in the regulation of synaptic plasticity and axonal growth in both CNS and PNS.	pDNM ‐gel with incorporation of Growth factor (NGF and VEGF)	PNI	**In vitro**: In Primary DRG neurons these scaffolds have shown increased axonal outgrowth, vascular formation, vessel‐nerve interaction, and SC proliferation/migration. **In vivo**: In adult male SD rats, supporting nerve tissue reconstruction and vascularization, which effectively reduced atrophy of the gastrocnemius muscles connected to the impaired sciatic nerves, ultimately led to significant electrophysiological transmission and locomotor functional recovery.	[25]
Chitosan	**Coated with alginate hydrogel encapsulated with neurotrophic factors** **Structure**: 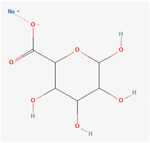 **Properties**: NT‐3 has the potential to differentiate the stem cells. Alginate hydrogels are used because they are involved in the vascularization of neural tissue engineering by bridging nerve gaps and guiding axons.	PCL/chitosan‐based electrospun nanofibrous scaffold (coated with alginate hydrogel encapsulated with neurotrophic factors)	SCI	**In vitro**: Improved differentiation and proliferation of Conjunctiva mesenchymal stem cells in the presence of NT‐3 growth factor. Increased biocompatibility and reduced inflammatory cell responses. High expression of neural‐specific genes and the manifestation of neuron‐like cell morphology. **In vivo**: PCL/chitosan/Alg nanofibers showed a mild inflammatory response when implanted in male Wistar rats. PCL/chitosan nanofibers implanted showed a moderate inflammatory response characterized by the presence of predominant lymphocytes, macrophages, and a few giant cells.	[146]
Collagen	**Cetuximab** **Structure**: 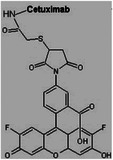 **Properties**: Enhanced differentiation of neurons and decreased astrocytic differentiation. Epidermal growth factor receptor signaling activation.	Collagen scaffold loaded with cetuximab	SCI	**In vitro**: Increase neurogenesis of the injury‐activated Neural stem cells at the lesion site. **In vivo**: Cetuximab could significantly increase neurogenesis of the injury‐activated NSCs at the lesion site. Implantation of collagen scaffolds loaded with cetuximab into lesion sites promoted neuronal and locomotion recovery of severely SCI dogs.	[147]

### 2.2 Biomaterials Used for Neurogenesis and Differentiation of Stem Cells

Fibrous scaffolds have been widely studied for neurogenesis via the use of stem cells, which have advantages such as increased attachment of cells to the scaffold, increased formation of neurites, and no inflammatory response. Fibers that are aligned support nerve cell proliferation, attachment, and differentiation.^[^ ^24^ ^]^ Hydrogels made of chondroitin‐4‐sulfate and alginate have been shown to mitigate the effects of neurological disorders and repair damaged nerves via increased neurogenesis. These hydrogels have been shown to increase intracellular signaling and conductivity. Specifically, upregulated expression of vascular endothelial growth factor receptor 2 (VEFGFR2), fibroblast growth factor 2 (FGF2), and TEK tyrosine kinase (TIE2), which are necessary for the remodeling and regeneration of nerves, has been reported.^[^ ^25^ ^]^ Here, we highlighted new biomaterials used for neurogenesis in preclinical studies (Table 1). Neural stem cells hold significant potential for regenerative medicine because of their self‐renewal ability. and *trans*‐differentiation abilities. The capacity of these cells to generate neurites, express neuronal proteins, and form synaptic connections with neighboring cells makes them more suitable for successful regeneration of CNS tissue.^[^ ^24^ ^]^ Owing to their superior cell adhesive abilities and high biocompatibility, natural biomaterials that consist of alginate (anionic polymer obtained from seaweed), collagen, gelatin (obtained from the hydrolysis of collagen), elastin (composed of glycine, proline, and isodesmosine amino acids), and chitin are frequently used as a 3D framework for the repair of nerve tissue.^[^ ^26^ ^]^ Bioactive molecules have been used to enhance the differentiation of stem cells into neurons and neurogenesis under conditions of neurological dysfunction. The structure and functional properties of these materials are highlighted in Table 1. It has been demonstrated that 3D collagen hydrogels aligned and directed by mechanical strain after gelation stimulate neuronal development more effectively than 2D cultures do, leading to elongated morphologies in both single‐cell neurons and nerve tissue explants.^[^ ^27^ ^]^ Nevertheless, owing to their poor mechanical durability, natural scaffolds have been reinforced with synthetic biomaterials to increase their mechanical strength. The ability to incorporate different neurotrophic factors for the repair of neurons has been made possible by the use of various synthetic biomaterials that have various functions, such as good mechanical durability, slow biodegradation, and low toxicity.^[^ ^26^ ^]^ PLGA is a synthetic polymer that is made from the polymerization of glycolic acid and lactic acid. It is frequently utilized as a biomaterial because of its low immunogenicity, biocompatibility, lack of toxicity, and mechanical ability. However, modifications are needed to improve its poor hydrophilicity and cell adhesion ability. PEG enhances the functionality of PLGA by facilitating cell adhesion and proliferation. PEG is well known for its hydrophilicity and anti‐inflammatory qualities. The proper orientation and connectivity of fibers can induce specific phenotypes in human neural cells, with significantly more myelin‐associated protein‐2‐positive cells in the PLGA‐PEG groups than in the PLGA alone group. Neural progenitor cell development toward mature cell types is enhanced as a result of the unique physiochemical characteristics of PLGA‐PEG.^[^ ^24^ ^]^ Furthermore, Yan et al. developed vertically aligned silicon nanowire (SiNW) arrays that preserve excellent cell viability and significantly enhance neural stem cell differentiation and proliferation. These cells exhibited markers for differentiation and proliferation at the mRNA and protein levels. The dynamic 3D structure of the SiNW array provides an optimal microenvironment for effective nutrient transport and mimics the natural extracellular matrix, which promotes NSC proliferation and differentiation.^[^ ^28^ ^]^

## 3 Application of Biomaterials in Nerve Tissue Injuries

**Figure 4:**
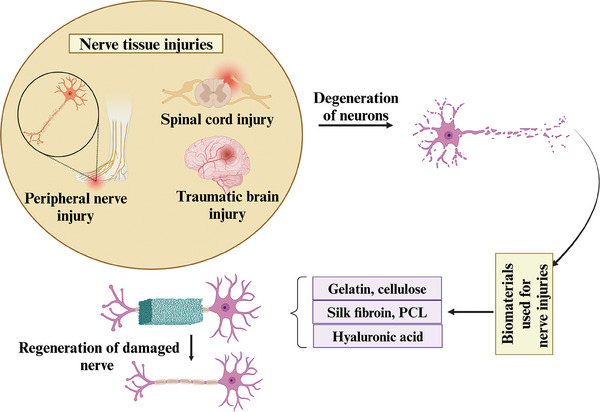
A comprehensive illustration of the different biomaterials utilized for the repair and regeneration of nerve tissue injuries.

Recently, the use of biomaterials has emerged as a promising therapeutic approach for neurological dysfunction, ranging from traumatic brain injuries to neurodegenerative disorders. These compounds have a significant effect on PNI and SCI. Biocompatible scaffolds and implants have been shown to promote tissue repair and regeneration, restoring damaged neural tissues, as shown in **Figure** **4**.

### 3.1 Peripheral Nerve Injury (PNI)

In animals and humans, PNI frequently results in severe and protracted functional and physical problems.^[^ ^29^ ^]^ The nervous system might sustain damage because of mechanical and thermal factors, leading to ischemia. Injury to the nervous system is characterized by the transfection of nerves, disruption of communication between neuronal cells, and disintegration of the blood–nerve barrier.^[^ ^6^ ^]^ According to the Sunderland classification, peripheral injuries can be categorized into 6 grades: neuropraxia (grade I), damage to the axons of the neuron (grade II), damage to the endoneurial tube (grade III), further damage to the perineurium (grade IV), complete transfection (grade V), and combined injuries (grade VI).^[^ ^30^ ^]^ Moreover, diseases of the motor and sensory nerves can be classified as axonotmesis, neurotmesis, or neurapraxia. When demyelination occurs without rupturing nerve continuity, there is transient interruption of the delivery of nerve impulses, known as neuropraxia, and this condition resolves completely after remyelination, which usually takes up to 12 weeks. The degeneration and rupture of axons are the hallmarks of axonotmesis. Regeneration takes place at a pace of 1 mm/day, extending from the site of damage to the peripheral organs.^[^ ^31^ ^]^ Neurotmesis is the total disruption in the continuity of a neuron caused by the lack of spontaneous regeneration of the injured neuron.^[^ ^31^ ^]^

Currently, sophisticated microsurgical methods or autologous nerve grafts with tensionless epineural sutures are used to repair damaged nerves and are preferred for treating PNI. Repair of peripheral nerves, however, does not guarantee a complete regain of functionality of the nerve. These findings suggest that the molecular and cellular mechanisms involved in traumatic PNI are not well addressed by microsurgical techniques.^[^ ^32^ ^]^ For the treatment of significant nerve damage, autologous nerve transplantation is the standard method. When peripheral nerve damage is severe, vascularized nerve transplantation is used to provide oxygen, blood flow, and nutrients to the damaged nerve. However, this process damages healthy nerve tissues since it often uses a nerve donor from an autologous sensory nerve.^[^ ^33^ ^]^ Autologous nerve grafting has several drawbacks, such as morbidity at the donor site, scarring, loss of sensory function, mismatch in the diameter of the nerve tube, and impaired sensation.^[^ ^34^ ^]^ To improve recovery following post‐traumatic PNI, several therapeutic approaches, including pharmaceutical (**Table** **2**), cell‐based, and electrical approaches, have been investigated, but none have been translated into clinical remedies devoid of adverse effects.^[^ ^32^ ^]^

**Table 2:** Clinical drugs and therapies for the treatment of neurodegenerative diseases (NDs) and neurological tissue injury (TI).

NDD/TI	Mechanism/Class	Drug/Therapies
**Clinical Drugs and therapies for Neurodegenerative disorders**		
Alzheimer's disease	Breakdown of acetylcholine	Rivastigmine, Donepezil, Galantamine, Aricept, Exelon
	Blocking of NMDA receptors affects the transmission of glutamate	Memantine
	Orexin receptor antagonist	Suvorexant
	Anti‐amyloid beta monoclonal antibody	Belsomra
Parkinson's disease	Antagonist for dopamine	Kynmobi, Neuupro, Requip XL, Mirapex
	Monoamine Oxidase	Eldepryl, Zelapar, Azilect
	Invasive surgical technique	Deep brain stimulation
	Treats motor fluctuations and dyskinesias	Levodopa‐carbidopa enteral suspension
Huntington's disease	Antipsychotic/inhibit dopamine receptor	Olanzapine, Risperidone
	Reversible inhibitor of VMAT‐2	Tetrabenazine
**Clinical Drugs and Therapies for Neurological Tissue Injury**		
Peripheral nerve injury (PNI)	Short gap PNI	Neurorrhaphy
	Promotes axonal regeneration	Phototherapy
	Calcium channel blocker	Nimodipine
	Treat peripheral neuropathies and postherpetic neuralgia	Gabapentin
Spinal cord injury (SCI)	Estrogen receptor modulator	Tamoxifen
	Antihistamine drug	Clemastine
	Early treatment for neuroprotective effect	Surgical decompression
Traumatic brain injury (TBI)	Surgical interventions	Decompressive craniectomy Kempe hemispherectomy incision Cisternostomy
	Complementary therapy	Phytotherapy Acupuncture
	Pharmacological therapy of traumatic brain injury	Corticosteroids Phenserine Calcium channel blockers Antioxidants Beta‐blockers Metformin Cerebrolysin Vitamin D

Several clinical studies have examined the use of scaffolds for the treatment of peripheral nerve injury. To this end, multiple scaffolds have been approved by the FDA, including neurotube, neurolac, neuroflex, neuromend, neurogen, neurowrap, and neuromatrix.^[^ ^35^ ^]^ 7,8‐Dihydroxyflavone, encapsulated in GelMA/SFMA (methacrylate gelatin and methacrylated silk fibroin), shows potential for treatment with complete functional recovery by boosting the remyelination of axons and producing a favorable milieu for regenerated nerves.^[^ ^36^ ^]^ In one study, cytokines and extracellular vesicles produced from M2 macrophages were encapsulated in a self‐assembled hydrogel of bionic peptide to create an environment resembling the immunological milieu. Regenerative cytokines such as VEGF, CCL‐20, CCL‐5, and IL‐10 were found to be highly expressed in conditioned media derived from M2 macrophages. This highlights the possibility of employing immune cell derivatives as bioactive materials for precise and gentle modulation of macrophage polarization. These findings also shed light on the importance of M2 polarization in tissue regeneration and the difficulties posed by disruptions in M2 function. Direct and minimal modification of M2 macrophages contributes to an efficiently controlled transformation rate in the polarization process and decreases immune reactions and adverse effects. This study presents a unique method that uses a self‐assembling peptide framework made of amino acids. It promotes the regeneration of long‐distance nerves in the periphery by facilitating the recruitment and transformation of M2 macrophages. M2 macrophages play important roles in the healing and integration of regenerated tissues;^[^ ^37^ ^]^ therefore, this approach is considered a treatment strategy for peripheral nerve damage.^[^ ^36^ ^]^ In another study, neural grafts that have been altered with the extracellular matrix produced by MSCs of human bone marrow were shown to improve the regeneration of nerves and stimulate the adhesion of cells while also directing the cells and temporally regulating the microenvironment.^[^ ^38^ ^]^ The regeneration of the nerve conduit resulted in functional recovery similar to that of an autograft. A large nerve gap of 15 mm was closed by intervention with the GelMA‐bFGF conduit in the rat model.

### 3.2 Spinal Cord Injury (SCI)

Statistics reported by the National Centre for Spinal Cord Injury in 2022 revealed that ≈78% of injuries related to the spinal cord were observed in males. Globally, the incidence of SCI varies from 3.6 to 195.4 cases per million.^[^ ^39^ ^]^ SCI is a debilitating condition characterized by damage to the spinal cord and neuropathological changes such as damage to neuronal cells and swelling. Thus, it causes deterioration of the extracellular matrix and results in scars on glial cells and cystic cavities.^[^ ^40^, ^41^ ^]^ Accident‐related SCIs can result from the central nervous system, which often experiences substantial stress. It causes white matter damage, disruption of the myelinated fiber tract, and myelopathy, which impairs the flow of motor and sensory information to and from the brain.^[^ ^42^ ^]^ It can be classified as thoracic or cervical depending on the site of the injury. Two complex phases make up the pathophysiology of SCI: the primary injury phase, characterized by muscular impairment, and the secondary injury phase, which disrupts the blood‒brain barrier and is characterized by inflammatory reactions, changes in neurotransmitter levels, and the infiltration of blood cells into the medullary tissues.^[^ ^42^ ^]^ In the limb just below the site of injury, scars in the glial cells and cystic cavities obstruct the regeneration of axons and transmission of electrical signals, causing long‐term motor impairments, autonomic dysfunction, and impairment of the senses. In addition to causing severe psychological and physical trauma to victims, SCI has a major financial impact on society. As a result, the prognoses of patients with SCI, prevention, and treatment have consequently gained substantial attention from the medical community.^[^ ^41^ ^]^

There is currently no therapy for SCI that can successfully restore the function of damaged tissue. Moreover, the poor reparative potential of nerve tissue coupled with the production of antiregenerative factors and persistent chronic inflammation leads to an impaired healing process. Neurodegenerative and neuroprotective approaches are currently used with an emphasis on stabilization, resuscitation, and specialized critical care. Early interventions, such as the administration of anti‐inflammatory drugs and surgical decompressions, have demonstrated the ability to decrease acute complications within 24 h of injury (Table 2).^[^ ^43^ ^]^ Multimodal care for SCI patients includes physical therapy and the prevention of complications to maximize the recovery of neurological function. Furthermore, regenerative treatments are being investigated extensively to improve clinical results by utilizing novel therapeutic approaches for neurological impairments.^[^ ^43^ ^]^

Recent developments in biomaterials, such as hydrogels derived from decellularized extracellular matrices, constitute promising platforms for nerve tissue regeneration and cell transport, especially in spinal cord regeneration.^[^ ^44^ ^]^ The incorporation of neurotrophic growth factor in silk fibroin/hyaluronic acid‐based hydrogels improved the regeneration of damaged neurons at the injured site and decreased the swelling and formation of the cavity.^[^ ^45^ ^]^ Similarly, the encapsulation of WAY‐316606 (a small molecule) in the PCL microfiber enabled the successful development of the composite hydrogel. Upon implantation in vivo, they were observed to fill gaps and prevent scarring of glial cells, increase motor function, and improve the regeneration of damaged neurons at the site of injury. Notably, the Wnt/β‐catenin pathway was triggered by the hydrogel encapsulating WAY‐316606, which promoted the development and differentiation of neuronal cells and the repair of damaged tissues in the spinal cord.^[^ ^46^ ^]^ Furthermore, a 3D gelatin scaffold improved the milieu at the injury site and decreased the accumulation of fibroblasts, allowing for the presence of neural stem cells, stromal cells, and Schwann cells. Consequently, this helped the injured spinal cord heal structurally in monkeys.^[^ ^47^ ^]^ PLA nanofibers coated with eumelanin have been shown to promote the proliferation of neural cells under in vitro conditions and have anti‐inflammatory properties. It has also been reported that they can reduce the generation of reactive oxygen species (ROS), block the expression of NF‐κB, and lower the expression of IL‐6.^[^ ^48^ ^]^

### 3.3 Traumatic Brain Injury (TBI)

TBI refers to a variety of mechanical forces, either acute or blunt, that cause hypoxia and vascular damage. This leads to the activation of glial cells and causes swelling, cell death, and loss of tissues.^[^ ^49^ ^]^ Studies suggest that TBI is a significant cause of disability, morbidity, and death in adults. Every year, more than 50 million individuals globally suffer from TBI.^[^ ^50^ ^]^ Both primary and secondary injury mechanisms play a role in TBI. Primary injuries occur immediately after the effect and include intraparenchymal and extraparenchymal hemorrhages, traumatic injuries to the axons, and cerebral edema. However, secondary injury has occurred over the years and includes disruption of the blood‒brain barrier, oxidative stress, mitochondrial dysfunction, swelling, cell loss, and excitotoxicity.^[^ ^51^ ^]^

The course of treatment for TBI varies depending on the extent of the injury and may include medications, surgical procedures, and cognitive therapy (Table 2).^[^ ^52^ ^]^ The tetracycline derivative minocycline reduces inflammation and apoptotic processes and is a pharmaceutically effective treatment for many disorders affecting the central nervous system. Strong anti‐inflammatory drugs for traumatic brain injury include fenofibrate, pioglitazone, and rosiglitazone, which are agonists of the peroxisome proliferator‐activated receptor (PPAR).^[^ ^53^ ^]^

A recent study demonstrated that hydrogels prepared from sodium alginate/collagen/stromal cell‐derived growth factor 1 can continuously release stromal cell‐derived growth factor 1, which promotes the survival and migration of MSCs in the bone marrow. It also helps to restore neurological functions by activating the PI3K/AKT/FAK pathway mediated by SDF‐1/CXCR4 in TBI. Hence, this method may be the most effective way to promote the healing of damaged tissues and regenerate against brain damage.^[^ ^54^ ^]^ However, hydrogels made of GelMA‐imid (imidazole) were loaded with human amniotic fluid‐derived MSCs and stromal cell‐derived growth factor 1 via the use of polydopamine nanoparticles as carriers. Under in vitro conditions, it stimulated the differentiation and migration of MSCs into nerve cells. It also reduces the number of Nissl bodies and decreases the size of the wound region while treating cryogenic brain injury.^[^ ^55^ ^]^ The chitosan scaffold was cross‐linked with genistein and loaded with a neurotrophic factor derived from the brain, resulting in the development of NSCs into neurons from the hippocampal region of rats, which can actively release neurotrophins and growth factors.^[^ ^56^ ^]^

## 4 Application of Different Biomaterials in Neurodegenerative Disorders

**Figure 5:**
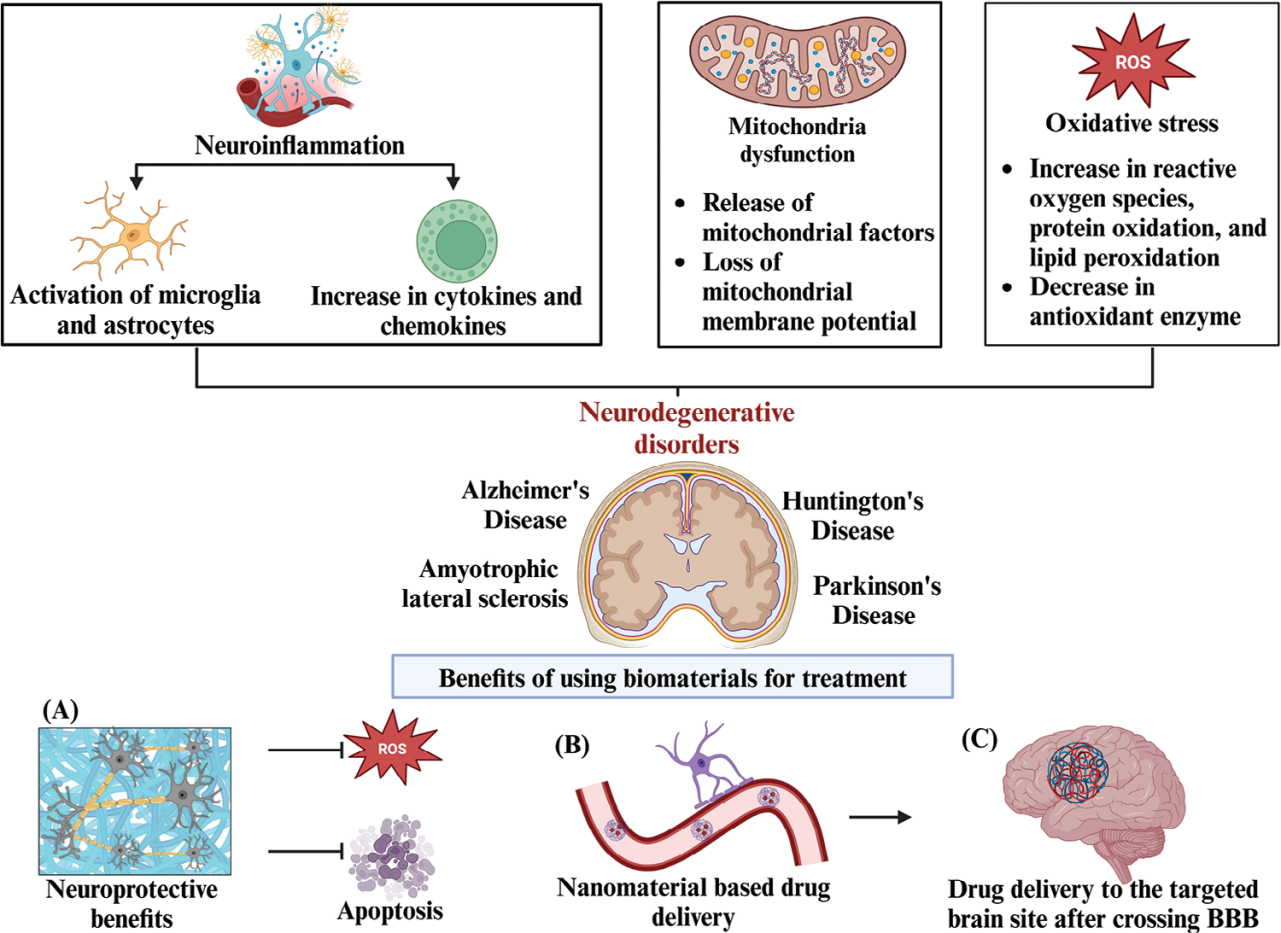
Schematic illustration of the biomaterials used in neurodegenerative disorders. A) Biomaterials have been shown to have neuroprotective effects by decreasing oxidative stress and inhibiting cell death. B,C) Nanomaterials have been shown to cross the blood‒brain barrier and reach the targeted site of the brain for sustained drug release to the targeted site.

In the context of NDs such as Alzheimer's disease and Parkinson's disease, biomaterials offer novel approaches for drug delivery systems and reduce the oxidative stress and cell death caused by NDs, as shown in **Figure** **5**. Hydrogels and nanoparticles, for example, enable the controlled and targeted release of therapeutic agents to affected regions of the brain, thus minimizing side effects and improving treatment efficacy. Biomaterials also play crucial roles in neuromodulation and neurostimulation therapies for conditions such as epilepsy and chronic pain. Electrodes and neural interfaces made from various advanced biomaterials facilitate precise and controlled stimulation of neural circuits, providing relief to patients who may not respond well to traditional treatments.

Furthermore, in the context of cerebrovascular disorders such as strokes, biomaterials have contributed to the development of innovative hemostatic agents and tissue‐engineered constructs. These advancements aim to minimize damage, promote tissue repair, and enhance recovery in individuals affected by cerebrovascular events. The versatility of biomaterials is exemplified in their applications across diverse neurological dysfunctions, highlighting their potential to revolutionize treatment approaches and improve patient outcomes. As research in this field continues to advance, integrating biomaterial‐based solutions into clinical practice holds immense promise for addressing the multifaceted challenges posed by neurological disorders.

### 4.1 Alzheimer's Disease (AD)

The predominant cause of dementia is AD, which is one of the major healthcare issues in our century. Worldwide, an estimated 40 million individuals are thought to be affected by dementia, and this figure is expected to double every 20 years until 2050.^[^ ^57^ ^]^ AD is the leading cause of dementia in people aged 60 and above. It accounts for 50% to 75% of dementia cases. Global data have shown that women are more likely to suffer from AD than males are, and the risk of AD increases with age.^[^ ^58^ ^]^ Memory loss is the most common symptom of age‐related disorders and is characterized by the gradual loss of cognitive ability. The medial temporal lobe of the hippocampus is implicated in memory and learning and is involved in processing spatial information.^[^ ^58^ ^]^ Fibrils of hyperphosphorylated tau protein and aggregates of Amyloid β 40 (Aβ40) are responsible for the primary pathogenic state of AD. Pathological manifestations occur far before symptoms appear.^[^ ^59^ ^]^ Early‐onset disease is extremely rare and is usually caused by genetics; therefore, it is classified as familial AD. An individual with Down syndrome who has an additional copy of the 21^st^ chromosome is very likely to develop early‐onset AD. Aβ protein, mostly seen in AD plaques, is produced when β‐secretases cleave the amyloid protein precursor sequentially. Additionally, the Tau protein becomes hyperphosphorylated and accumulates to form neurofibrillary tangles. Despite the lack of clarity on the interaction of Aβ and tau proteins, the amyloid cascade theory states that elevated Aβ levels precede tau aggregation, often in the cortical area.^[^ ^60^ ^]^

AD currently has four FDA‐approved therapies (Table 2), all of which are principally linked to two molecular pathways involving the accumulation of p‐tau and the Aβ peptide. However, none of these drugs stop the progression of the disease, thus emphasizing the need for other therapeutic approaches.^[^ ^61^ ^]^ Rivastigmine, donepezil, and galantamine block the acetylcholinesterase (AChE) enzyme, whereas memantine acts as an N‐methyl‐D‐aspartate receptor antagonist. Nevertheless, with the administration of these drugs, the symptoms improved only slightly. They do not stop the deterioration of cognitive function caused by neuronal cell death and brain shrinkage.^[^ ^62^ ^]^ In preclinical and clinical investigations, several therapeutic approaches targeting these pathological processes have failed owing to the short half‐life of the drugs, low bioavailability, inadequate penetration across the blood‒brain barrier, and poor uptake by the cells. Therefore, to delay the onset of AD, disease‐modifying therapies are necessary.^[^ ^59^ ^]^ Since the blood‒brain barrier restricts the number of therapeutic drugs that may enter the CNS, the availability of drugs in the CNS is limited. To combat this issue, active chemicals are bound to nanocarriers such as micelles, liposomes, and polymeric and lipid nanoparticles because of their high permeability across the blood‒brain barrier. This facilitates faster and more effective transport of the drug to the CNS. Another approach involves the implantation of biodegradable biomaterials loaded with drugs, including microparticles and nanoparticles. This provides a way to transport medications precisely to the intended site of action, perhaps encouraging the regeneration, repair, or preservation of injured CNS tissue.^[^ ^63^ ^]^

The implantation of gelatin/PEG nanocomposites encapsulated with magnesium hydroxide significantly improved the nonspatial and spatial memory skills of the rats. The scaffold was better associated with AChE enzymes and phytochemicals, thus indicating the stability of the AChE phytochemical complex. Nanocomposite scaffolds even exert neuroprotective benefits through their ability to reduce oxidative stress in neuronal cells, modulate brain inflammation, and increase antioxidant activities.^[^ ^64^ ^]^ Pressurized gyration is used to load vitamin B‐12 and donepezil into the nanofibers of PCL/PVP. Quick and sustained release of drugs was observed from the nanofibers, which also displayed a stable structure and shape that made them biocompatible with the drug. These nanofibers encapsulated with the drugs protected the cells against Aβ‐induced toxicity toward the neuronal cells and reduced BACE‐1 and APP gene expression while increasing ADAM‐10. These developed nanofibers are a viable substitute for transdermal drug delivery for AD treatment. Another study reported that PLGA‐PEG nanoparticles loaded with curcumin coupled with the B6 peptide led to decreased tauopathy in APP/PS1 mice. These results highlight the improvement in nanoparticle distribution to the brain with PLGA‐PEG‐B6 and imply that the use of PLGA‐PEG‐B6/Cur nanoparticles might become a viable therapeutic approach in the future for treating AD.^[^ ^65^ ^]^

### 4.2 Parkinson's Disease (PD)

PD is a degenerative neurological condition characterized by the deterioration of dopaminergic neurons, resulting in diminished dopamine levels and eventually leading to neuronal death. It impacts the neural system, which aids in central, enteric, autonomic, and visual functions.^[^ ^66^ ^]^ PD is characterized by tremors, rigidity of muscles, bradykinesia, and depression.^[^ ^67^ ^]^ A unique pathological feature of PD is the formation of Lewy bodies, consisting of ubiquitin and α‐synuclein, which are deposited intracellularly in the neuronal cell body. Ubiquitin and α‐synuclein accumulate through disturbed vesicular trafficking and mutation in the genome.^[^ ^68^ ^]^ In addition to dementia, multiple system atrophy, and autonomic failure, disorders involving impaired α‐synuclein are collectively referred to as synucleinopathies.^[^ ^69^ ^]^ α‐Synuclein is a native unfolded protein of 140 amino acids that is generally involved in synaptic activity and intracellular trafficking. However, the precise physiological function of this protein remains unknown.^[^ ^70^ ^]^ Toxic oligomers of α‐synuclein disrupt calcium levels between organelles, affecting cellular functions such as trafficking to the endoplasmic reticulum, functioning of mitochondria, degradation pathways, and interactions between the mitochondria and endoplasmic reticulum.^[^ ^71^ ^]^

PD is a complicated disease to treat because there are various PD subtypes with a wide range of nonmotor and motor symptoms. There is an increase in the use of invasive interventions such as deep brain stimulation and drug pumps. These medical therapies are tedious and expensive.^[^ ^72^ ^]^ Although modern therapies, including approved drugs (levodopa, selegiline, Istradefylline, etc.) and combination therapies, frequently improve motor function, as highlighted in Table 2, they are also linked to significant adverse effects resulting from nonphysiological release and dopamine transport.^[^ ^73^ ^]^ Owing to their physical and chemical properties, most of the treatments have limited bioavailability, poor pharmacokinetics, and an inability to permeate across the blood‒brain barrier.^[^ ^74^ ^]^ Cognitive dysfunctions, dyskinesia, and variations in response to therapy are common in PD patients, and these symptoms have a significant negative influence on their quality of life. Improved therapies, such as those that change the fate of the disease, are needed, but the absence of reliable biomarkers and preclinical disease models makes drug development challenging.^[^ ^73^ ^]^

Tissue engineering is a novel and advanced technology that aims to alleviate the limits of conventional treatments for PD. A drug delivery system for the brain was developed by conjugating PEG‐PLGA nanoparticles with lactoferrin. PEG‐PLGA nanoparticles can easily cross the brain's barrier and are biodegradable. In vitro, and in vivo, lactoferrin is transported through the lactoferrin receptor expressed by neural cells and penetrates easily through the blood‒brain barrier, while the PEG chains impart longer residence durations in the body. Another study showed that nanoparticles loaded with urocortin decreased the formation of striatum lesions caused by 6‐hydroxydopamine.^[^ ^75^ ^]^ Injectable electroconductive hydrogels based on chitosan, a natural polymer, improved the proliferation and viability of neural stem cells along with rapid self‐thinning and self‐healing properties. These compounds exhibited anti‐inflammatory and ROS‐scavenging properties in vivo. The hydrogels injected into a PD model in rats significantly improved motor function. This material might be a viable biomaterial for the treatment of PD and neuroprotection,^[^ ^76^ ^]^ encapsulating dopaminergic progenitors derived from human pluripotent stem cells in a biocompatible matrix of hydrogels tailored to support these cells. The incorporation of laminin and encapsulation of neurotrophic factors derived from glial cells in this hydrogel enhances motor deficits in experimental models, and A9 neuronal cell counts are markedly increased by 51%.^[^ ^77^ ^]^ These findings are especially promising because earlier attempts to transport dopamine across the blood‒brain barrier have failed. As documented in several research papers, further investigations have investigated the use of hydrogels, collagen, and synthetic biomaterials such as PGLA for dopamine delivery.^[^ ^78^ ^]^

### 4.3 Amyotrophic Lateral Sclerosis (ALS)

Jean‐Martin Charcot initially described ALS as a motor neuron disease in 1869, but it is now understood to be a multisystem ND with genetic, clinical, and pathological variability.^[^ ^79^ ^]^ Upon onset, ALS progresses quickly and usually results in death two to three years after symptoms appear. ALS results in leg and arm weakness, speech defects, and swallowing difficulties before progressing to paralysis, which is a common clinical manifestation. Cramping, fasciculation without muscular weakness, emotional instability, cognitive problems, weight loss, and respiratory failure (type 2) are among the lesser prevalent manifestations of ALS. Lower motor neuron defects manifest as fasciculations, muscular atrophy, and weakness, whereas the altered function of higher motor neurons causes spasticity and rapid deep reflexes.

The diagnosis of ALS is made solely on the basis of clinical characteristics, as there are no known radiological or serological indicators of the disease.^[^ ^80^ ^]^ Proteinopathy caused by TAR DNA‐binding protein 43 (TDP‐43) is present in ≈97% of cases of ALS. TDP‐43 is lost from the nucleus and aggregates in the cytoplasm to form compact‐like structures. Although distinct aggregates of cytoplasmic proteins are observed, ALS linked to mutations in the SOD1 and FUS genes does not result in TDP‐43 proteinopathy. This finding indicates pathogenic variety. The most prevalent genetic subtype has p62‐positive protein aggregates caused by pathogenic dipeptide repeat proteins (DPRs), TDP‐43 mislocalization, and hexanucleotide GGGGCC expansions in the C9orf72 gene.^[^ ^81^ ^]^

ALS has the longest pharmaceutical pipeline of any neuromuscular condition, yet no drug can stop or reverse disease development. To improve the ALS clinical environment and accelerate the release of novel medicines into the market, dynamic and cooperative clinical trial networks still collaborate with government agencies, businesses, and the patient community.^[^ ^82^ ^]^ For the treatment of ALS, edaravone and riluzole are the two medications that have some positive effects on the disease condition, as highlighted in Table 2.^[^ ^83^ ^]^

It has been reported that silica nanoparticles loaded with pioglitazone and leptin have enhanced motor function and can cross the brain barrier to treat ALS in TDP‐43A415T mice.^[^ ^84^ ^]^ In another study, gold nanoparticles encapsulated with hypoxia‐inducible factor presented a novel strategy to halt the development of ALS by enhancing the self‐renewal and proliferation of ependymal stem progenitor cells in mice.^[^ ^85^ ^]^ By stimulating the retinoid pathway, retinoid‐activating nanoparticles increased motor function, extended longevity, and provided neuroprotection in the SOD1G93A mouse model, indicating a unique platform for retinoid‐mediated ALS therapy.^[^ ^86^ ^]^ Chitosan and transferrin nanoparticles loaded with riluzole better absorbed the drug via the nasal route. Pharmacodynamic assessments revealed that these compounds could be useful as antianxiety treatments. With the additional benefit of transferrin having high target effectiveness, the produced nanoparticles demonstrated neuroprotective effects by reversing the toxicity generated by haloperidol. This work demonstrated the great promise of riluzole loaded in chitosan nanoparticles and riluzole loaded in transferrin and chitosan nanoparticles for intranasal administration, particularly for CNS illnesses. Nevertheless, further preclinical and clinical research is needed before these compounds can be made commercially available.^[^ ^87^ ^]^ A 3D nanobiohybrid hydrogel implanted with carbon nanotubes with a functionalized carboxyl group and neural spheroids was used to construct a biosensor system for neuromuscular junctions. This technology efficiently tracks muscle healing and mobility for medication assessment when cultured along with a 3D muscle bundle. It improved neuromuscular junction development by increasing the number of neurites and their connection to the muscle bundle. Additionally, by encouraging neural spheroid neurogenesis and increasing hydrogel conductivity, the incorporation of carboxyl‐functionalized carbon nanotubes into the hydrogel increased neuromuscular junction development.^[^ ^88^ ^]^

### 4.4 Huntington's Disease (HD)

As a proteinopathy, HD shares traits with polyglutamine disorders, ALS, PD, and AD. While HDdoes not manifest until mid‐adulthood, early events such as the effect of the mutated Huntingtin protein on the proliferation of neural cells and neuron development may pave the way for the disease later.^[^ ^89^ ^]^ It is estimated that 5–12 per 100 000 people suffer from HD in the UK, making it the most common ND in the West.^[^ ^90^ ^]^ Motor impairment, mental problems, and cognitive decline are the characteristics of HD. Mutation of the huntingtin protein and neurotoxicity cause motor symptoms such as stiffness and chorea, which cause atrophy in different parts of the brain. Juvenile onset and late onset are both included in the clinical spectrum of HD. Children who have certain genetic markers may experience delayed physical development, and certain symptoms include discomfort, sleeplessness, itching, and psychosis.^[^ ^91^ ^]^ HTT is prone to misfolding because of the high number of CAG repeats, which causes the expansion of the polyglutamine present in the N‐terminal region of the protein. Insoluble aggregates caused by misfolding are discovered in the cytoplasm of damaged neurons as well as in the nucleus, where they form intranuclear inclusions. The accumulation of these aggregates results in significant shrinkage, apoptosis, and cellular malfunction in affected brain regions. The hippocampus, cerebral cortex, cerebellum, hypothalamus, and some brainstem and thalamus nuclei also exhibit atrophy.^[^ ^92^ ^]^

The focus of HD is on managing chorea symptoms and treating cognitive and mental health issues. The FDA‐approved drugs for the treatment of HD include deutetrabenazine and tetrabenazine, which successfully diminish chorea in HD patients, as highlighted in Table 2.^[^ ^93^ ^]^ Studies have indicated that the best treatment for patients with HD requires an interdisciplinary approach that includes neurologists, psychiatrists, and physical and speech therapists.^[^ ^94^ ^]^ HD has no known cure, and the therapies that are currently available are just symptomatic. Novel treatment approaches that can stop or postpone neuronal loss and address the underlying cause of HD are urgently needed.^[^ ^95^ ^]^

Researchers have discovered that selenium nanoparticles can alleviate behavioral dysfunction and reduce neuronal cell death. Treatment with selenium nanoparticles decreased the expression of HAD mRNA at the genomic level and decreased the aggregation of HTT and the levels of reactive oxygen species.^[^ ^96^ ^]^ A hybrid of g7 nanoparticles that were encapsulated with a relatively high concentration of cholesterol was developed. This hybrid‐g7‐NP‐chol is absorbed and travels to many brain cell types. It builds up over time and can release cholesterol, making it available for use by neurons. Moreover, these nanoparticles breakdown quickly in plasma and peripheral tissues without causing inflammatory reactions. Finally, they emphasized the usefulness of using cholesterol as a model medication to specify NP‐based delivery systems.^[^ ^97^ ^]^ Carbon nanotubes, graphene, and fullerenes are examples of carbon‐based nanomaterials that have attracted interest in nanomedicine because of their exceptional physicochemical characteristics. Fullerenes are electron deficient and therefore show strong antioxidant capabilities, thereby reducing the concentration of oxygen free radicals. They in turn inhibit the production of amyloid proteins and antagonistically inhibit glutamate receptors as well.^[^ ^98^ ^]^ Graphene has many benefits, including high surface areas, low toxicity, controlled drug release, and remarkable mechanical strength. Through the reduction and clearance of aggregated HTT protein, graphene functionalization represents a promising approach to prevent the progression of HD.^[^ ^99^ ^]^

### 4.5 Cerebral Stroke

Stroke is a severe medical emergency characterized by a disruption in blood flow to the brain, which leads to the deprivation of oxygen and nutrients in brain cells, resulting in cell death.^[^ ^100^ ^]^ Conversely, hemorrhagic stroke occurs when a blood artery bursts, causing internal bleeding in the brain. Factors such as elevated blood pressure, damage to the walls of blood vessels, or aberrant connections of blood vessels may cause this rupture.^[^ ^100^ ^]^ Strokes can lead to a variety of cognitive and motor defects, including depression and dementia. Approximately 16 million individuals worldwide suffer from strokes every year, resulting in a significant financial impact on society.^[^ ^101^ ^]^ The degree of permeability of the blood‒brain barrier is elevated when cerebral blood flow is disrupted, triggering a series of pathological cascades that include neuroinflammation and oxidative stress. Thus, following a stroke, enhanced permeability of the blood‒brain barrier linked to cerebral ischemia‒reperfusion may worsen clinical outcomes and cause long‐term impairment.^[^ ^102^ ^]^

FDA‐approved stroke treatments mostly aim to promote reperfusion to address immediate brain damage. These techniques have shown marked effectiveness in clinical trials and involve direct lysis of the blood clot, endovascular stents, and tissue plasminogen activators. Although rehabilitation modalities such as physical, speech, or occupational therapy are designed to increase patient mobility, their efficacy in promoting recovery after stroke has been limited. Currently, no known medical treatment promotes the healing and restoration of damaged tissues.^[^ ^103^ ^]^ Intravascular intervention treatment, such as endovascular thrombectomy, can be employed as a substitute or alternative therapy at risk of early vessel reocclusion. Compared with tissue plasminogen activator treatment alone, alternative therapy can increase the duration of treatment to 12 h and enhance vascular function and remodeling in patients. However, the duration of the therapy substantially influences the effectiveness of this intravascular intervention, given that a mere 30 min extension beyond the designated period might markedly diminish the patient's functional independence. Since less than 10% of stroke patients are suitable for this treatment and only regional stroke centers with neurological intervention skills can conduct this procedure, current endovascular therapies remain difficult to generalize.^[^ ^50^ ^]^

A study has shown that regeneration of damaged brain tissue may be induced by implanting a hydrogel made of extracellular matrix (the basement membrane and tunica propria of the porcine urinary bladder). After 90 days of implantation of the 4 mg ml^−1^ hydrogel, which was composed of an extracellular matrix of porcine urinary bladder matrix, an increase in the percentage of mature neural cells was evident in the residual scaffolding. Therapeutic investigations appear to be a potential route for *in situ* engineering of brain tissue via the use of inductive biomaterials.^[^ ^104^ ^]^ Furthermore, the electrochemical, mechanical, and electrical properties of the hippocampus are mimicked by a pluronic‐chitosan/aniline‐pentamer hydrogel loaded with VEGF, an angiogenic factor. The long‐term release of VEGF significantly reduces the infarct size in an ischemic rat model while improving hippocampus‐dependent memory and learning.^[^ ^105^ ^]^ In another study, a hyaluronic acid‐based hydrogel loaded with neural stem cell‐derived exosomes was used to treat ischemic stroke. It preserves the biological activities of exosomes, thus promoting cell proliferation, anti‐inflammatory effects, and angiogenesis in the ischemic area, thereby enhancing neurological function and cerebral infarction. This research indicates that the topical injection of this hydrogel is a promising approach for clinical application in the treatment of ischemic stroke.^[^ ^106^ ^]^ Dl‐3‐n‐butylphthalide loaded in ultrasmall cerium oxide nanoparticles was prepared and has shown remarkable free radical scavenging properties. These nanoparticles have good potential for use in clinical settings for the treatment of ischemic stroke, as they effectively decrease oxidative stress, retain mitochondrial function, decrease blood‒brain barrier damage, and promote nerve healing.^[^ ^107^ ^]^ A sericin scaffold was prepared and doped in carbon nanotubes that could stimulate the neural development of mesenchymal stem cells derived from bone marrow. They have photoluminescence and programmable shape memory capabilities. This scaffold demonstrated the viability and potential for treating severe ischemic strokes through extensive characterization, mathematical modeling, molecular and cellular data, imaging techniques, and in vivo stroke models. This finding highlights the novel shape of memory material for clinical application in brain tissue repair.^[^ ^108^ ^]^

## 5 Techniques for the Treatment of Neurological Disorders

The treatment of neurological disorders mostly involves the diffusion of various drugs across the blood‒brain barrier. The safe and suitable distribution of drug molecules to the brain is an exceptional aim for attaining the best‐targeted drug treatment for various neurological disorders.^[^ ^109^ ^]^ Designing a drug delivery system requires a thorough understanding of the properties of the biomaterials, such as their mechanical properties, biocompatibility, hydrophilicity, surface charge, and biodegradation. Additionally, understanding how biomaterials behave at different temperatures and pH values is crucial.^[^ ^110^ ^]^ Novel drug delivery approaches are favored when bioabsorbable and biodegradable polymers, especially hydrogels, nanoparticles, and smart biomaterials, are used. In the form of implants, these biomaterials provide a secure framework for drug administration without affecting the body of an individual.^[^ ^111^ ^]^

### 5.1 3D Bioprinting of Neural Tissue

**Figure 6:**
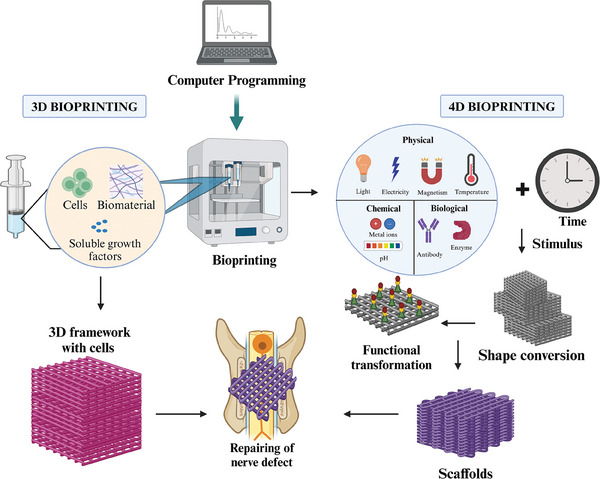
An overview of the bioprinting process. This includes both 3D and 4D bioprinting techniques, where 3D bioprinting involves creating complex tissue structures layer by layer, and 4D bioprinting incorporates time as an additional dimension, allowing printed structures to change shape or function in response to environmental stimuli.

3D printing involves a variety of flexible additive manufacturing processes that are capable of precisely creating complex 3D structures. It offers significant advantages in terms of design, reliability, mass customization, and compatibility with different materials (**Figure** **6**). On a computer‐controlled platform, cured printed materials are used to build a 3D structure layer by layer under the guidance of digital models. Using methods such as extrusion, inkjet, and stereolithography, 3D printing has been developed over the last century.^[^ ^112^ ^]^ Rapid prototyping approaches are used in 3D bioprinting, also referred to as additive manufacturing, and are used to print cells along with growth factors and various biomaterials layer by layer. Artificial organs and tissues produced via this technique closely resemble native tissues. Using 3D modeling software such as computer‐aided design (CAD) or computer tomography (CT) scan pictures, 3D bioprinting produces solid or gelatinous designs. The components of a standard 3D bioprinting system include computers; bioprinting ingredients/inks; an X‐, Y‐, and Z‐axis drive device; and a 3D modeling program.^[^ ^113^ ^]^

A recent study created a scaffold of live neurons via 3D bioprinting approaches that resembled the native spinal cord both macroscopically and microscopically by using bioink, which contains chitosan/hyaluronic acid/Matrigel. It facilitates the regeneration of damaged neurons and their connectivity, allowing quick restoration of locomotive function. This work revealed that 3D bioprinted scaffolds loaded with NSCs may be used for in vivo healing of SCI. These findings suggest that neural tissue engineering and regenerative medicine, which involves injury to the spinal cord, may benefit from these developments shortly.^[^ ^114^ ^]^ Furthermore, a conductive hydrogel has been created via this technique to systematically administer electrical stimulation and facilitate neuronal development. Interaction with dorsal root ganglion cells revealed that the conductive hydrogel had improved electrochemical characteristics and had less harmful effects than the other hydrogels did.^[^ ^115^ ^]^ Research is also being conducted on nanoparticles made up of metals such as silver and gold for the regeneration of specific damaged tissues.^[^ ^116^ ^]^ High‐modulus structural tissues have been developed with significant advancements in additive biomanufacturing, namely, 3D bioprinting. However, the dearth of chemically and physically biomimetic neural bioinks has made biofabrication of brain tissues difficult. This drawback can be addressed by developing innovative bioinks for 3D bioprinting of soft and free‐standing brain tissues. Bioinks with natural polymers such as gelatin and hyaluronic acid can be blended with synthetic polymers such as PF127 to mimic the extracellular matrix while attaining the desired rheological properties for 3D bioprinting. Thermally gelling inks with Herschel–Bulkley rheological behavior are created by mild cross‐linking based on thiol–catechol chemistry, which creates soft, free‐standing neural tissues. The resultant hydrogels had remarkable cell survival and moduli resembling those of the native brain tissue extracellular matrix.^[^ ^117^ ^]^

### 5.2 4D Bioprinting for Neural Tissues

An addictive manufacturing technique called 4D bioprinting can construct living tissues that dynamically alter themselves in response to predefined stimuli by stimulating physiological processes such as compression and stretching of these tissues. In 4D bioprinting, time is the fourth dimension (Figure 6). The characteristics of the printed products change over time in response to certain stimuli, exhibiting built‐in self‐assembly characteristics aided by cues contained in the printing and design material.^[^ ^118^ ^]^ Since they can be reverted to a “temporary‐permanent” state, shape memory polymers are very advantageous for less invasive surgical delivery. They allow for the activation of the *in situ* shape, which diminishes trauma and improves patient comfort while fostering a smooth transition between the injury site and the scaffold.^[^ ^119^ ^]^ Tissue engineering advances with a wide range of alternative biomaterials have sparked 4D bioprinting research for regenerative medicines, especially intricate tissue scaffolds.^[^ ^120^ ^]^ Two kinds of programmable microstructure trisegment nerve conduits were successfully printed via 4D printing using zein protein (derived from plants). By having tunable pore structures, these nerve conduits provide exact control over the degradation rate of nerve tissues and augment nerve regeneration. Regeneration of damaged nerves is more effectively facilitated by conduits that have been constructed with slower deterioration at the center and rapid degradation at both ends.^[^ ^121^ ^]^ Currently, 4D bioprinting in nerve tissue engineering is focused mostly on how the cells in the scaffold react to external stimuli, with relatively little research on how the scaffold itself evolves. In 4D bioprinting, shape memory properties might be included in nerve conduits in the future. These nerve conduits should be able to self‐adjust with stretching or bending action after they are implanted into human joints, nerves, or any other location to increase longevity.^[^ ^112^ ^]^

### 5.3 Neuromodulation and Brain‒Machine Interfaces

A novel approach for helping people with sensorimotor deficiencies is the combination of brain‒machine interfaces with neuroprostheses, which is an innovative method. Brain‒machine interfaces are devices that use individual brain activity to identify motor intent and convert it into movements. This allows the user more control over external devices, such as robotic arms or computer cursors. Neural activity linked to desired movement trajectories may be identified even without actual movement, which is the foundation of brain‒machine interfaces. Different recording techniques offer variable resolutions and accuracy, typically necessitating a compromise between invasiveness and information transmission quality. These activities can be converted into executable motor instructions via multichannel brain signal recordings and sophisticated algorithms.^[^ ^122^ ^]^ Advances in nanotechnology, nanomaterials, and molecular communications will enable synaptically interactive BMIs that perform better than existing technologies by enabling fine‐grained control over brain circuits.^[^ ^123^ ^]^

### 5.4 Bioelectronic Medicines

**Figure 7:**
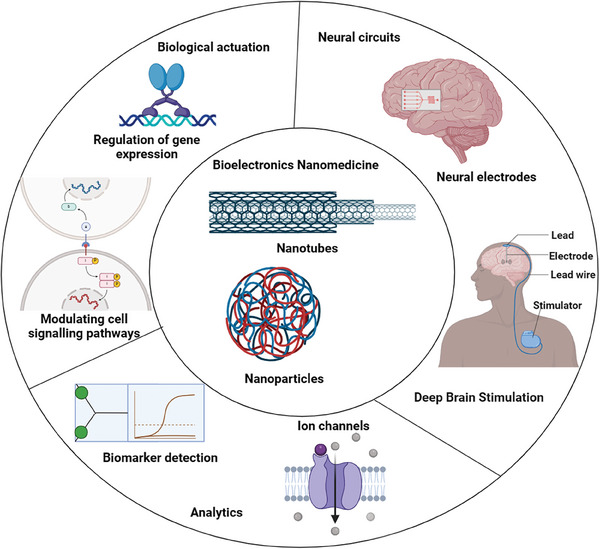
Bioelectronic nanomedicine is a vast area in which various electronic devices have been used for the treatment and monitoring of neurological disorders.

Bioelectronic medicine is a minimally invasive method rooted in neurology, molecular medicine, and biomedical engineering.^[^ ^124^ ^]^ Electric stimulation‐based treatment, as highlighted in **Figure** **7**, has been shown to accelerate the regeneration of native functional tissues.^[^ ^125^ ^]^ Notably, electrical impulses are sent by electrogenic cells, such as cardiac, musculoskeletal, and brain cells.^[^ ^126^ ^]^ Cell processes inside tissue can be engineered and manipulated via the use of computational tools combined with biocompatible scaffolds and electronic components such as transducers, electrodes, and transistors.^[^ ^125^ ^]^ Thousands of people have utilized deep brain stimulation since it was initially authorized in 1997 for the treatment of tremors in PD. OCD, depression, pain, Tourette syndrome, and AD are other possible deep brain stimulation targets. Another frequent target is the spinal cord, especially regarding treating chronic pain. In August 2018, the FDA authorized Nevro Corp's Senza II Spinal Cord Stimulation System. This device implants a battery‐operated generator beneath the skin to deliver electrical pulses to the spinal cord to treat persistent, uncontrollable pain in the trunk or limbs.^[^ ^127^ ^]^

Cochlear implants are effective neural prostheses that can aid patients with hearing impairment. In this technique, sound waves are sent directly to the brain, avoiding the cochlea, by positioning a series of electrodes at locations along the auditory nerve. Technological developments in bioelectronics have facilitated the application of complex techniques in clinical settings. These techniques include the use of vagus nerve stimulation to treat chronic conditions such as migraines, headaches, rheumatoid arthritis, and epilepsy, as well as brain stimulation therapy for PD.^[^ ^125^ ^]^ Recently, bioelectronic therapy is effective in substituting or completely replacing drug‐based therapies, which frequently have negative side effects and lack site specificity. Despite its enormous promise, the durability of the interactions between the surfaces of electrodes and tissues is a key obstacle in bioelectronics. Persistent scarring and inflammation are frequently the outcomes of limited biocompatibility, mechanical mismatch at the electrode/tissue junction, and significant material interface corrosion, which can severely affect device performance. To improve long‐term implantation without triggering tissue reactions and enabling integration into native tissues, research is now focused on producing biocompatible materials.^[^ ^128^ ^]^

### 5.5 Innovations in Monitoring and Diagnostics

Nanotechnology has emerged as a potential strategy to overcome obstacles in the early diagnosis of neurological disorders^,[^ ^129^ ^]^ which can be accomplished by targeting the shortcomings (gaps) in spatial resolution and blood–brain barrier penetration technology. The restoration of sensory and motor functions depends on neural interfaces. These neural interfaces (NIs) are critical components of brain recording systems and neuromodulation. Nevertheless, an imbalance between brain tissues and NIs may trigger immune responses that result in the scarring of glial cells and the death of neural cells. Neuroinflammatory responses further impair the stability and long‐term functionality of neural interfaces.^[^ ^130^ ^]^ To overcome these challenges, several ideas have been proposed for improved, flexible, battery‐free biointegrated NIs. New biomaterials at the brain device interface can enhance the spatiotemporal resolution of neural circuit probing with less invasive methods.^[^ ^130^ ^]^

Several nanomaterials have been shown to enhance specificity and sensitivity in biosensing and imaging, opening the door for better neurological diagnostics. These materials include carbon‐based nanomaterials, quantum dots, magnetic nanoparticles, plasmonic nanomaterials, and others.^[^ ^129^ ^]^ Owing to the increasing demand for the diagnosis and treatment of neurological disease, there is an increase in the need for sophisticated biomaterial development. Magnetic, electroactive, and photoactive materials are examples of functional biomaterials that are gaining popularity for their ability to react to external stimuli, offer structural support for damaged nerves, and encourage the proliferation of nerve cells and regeneration of damaged tissues.^[^ ^131^ ^]^

Presently, carbon and graphene nanotubes are the most well‐known forms of carbon nanostructures. These materials have been extensively studied because of their remarkable optical properties, mechanical toughness, and electrical and thermal conductivity. Carbon nanotubes have been demonstrated to stimulate neural development, survival, proliferation, and neurotransmission. Future neuroprosthetics may use carbon‐related compounds that use the growth of neural cells over nanostructures based on graphene to increase the development of neurons and signal transduction. Researchers are still investigating whether it helps in tissue regeneration and brain function following injury, despite the initial interest in its use as a scaffold for tissue engineering. Carbon nanofiber‐based nanoelectrodes created in partnership with the Mayo Clinic are utilized as instruments for neurochemical stimulation and monitoring, demonstrating their capacity to measure neurochemical concentration levels.^[^ ^132^ ^]^ Using a thermal drawing technique, biodegradable PLLA fibers with exceptional mechanical flexibility and optical clarity were developed. These fibers are used in vivo as optical neural interfaces to enable intracranial light supply and detection for deep brain fluorescence sensing and optogenetic interrogation.^[^ ^133^ ^]^

## 6 Clinical Trials of Biomaterials for Neurological Disorders

Different types of biomaterials have been used in preclinical trials. In vitro and in vivo, these biomaterials have been shown to increase the regeneration of damaged neurons. Clinical trials using different scaffolds have led to several new directions in the developing fields of nerve tissue engineering in recent years (**Table** **3**). Humanitarian device exclusion (HDE) research was conducted on people with thoracic AIS to assess the safety and potential benefits of the poly(lactic‐co‐glycolic acid)‐b‐poly(L‐lysine) scaffold (“Scaffold”). Compared with open spine surgery, traumatic spinal cord damage is the standard therapy at the neurological level of injury at T2–T12. The purpose of the scaffold is to support individuals between the ages of 16 and 70 years who have been diagnosed with SCI. In these cases, open spine surgery, such as laminectomy or spine stabilization, is advised as a possible course of treatment because it provides access to the injured spinal cord dura. During open spine surgery, the scaffold should be inserted into a cavity at the location of the spinal cord contusion. The scaffold is designed for use in cases of recent (within 7 days) SCI that do not entail total cord‐severing or penetrating damage. Six months after ferumoxytol treatment, ClinicalTrials.gov measured the gradient‐echo T2‐weighted signal change in the globus pallidus, which is a known brain iron reservoir. This signal is produced from an MR sequence sensitive to paramagnetic substances such as iron. The following are secondary outcome measures: 1) the quantity, location, and qualitative morphology of MS lesions enhanced by ferumoxytol, gradient‐echo phase, and gadolinium, as well as the evolution of these lesions; and 2) quantitative estimates of the change in iron concentration, as determined by measuring the R2 (=1/T2) relaxation rate within MS lesions, normal‐appearing white matter, normal‐appearing gray matter, and other iron‐rich brain regions before and following ferumoxytol injection (ClinicalTrials.gov).

**Table 3:** Various biomaterials in clinical trials in various countries for different neurological disorders.

Scaffold used	Neurological disorder	Phase of clinical trials	Location	Clinical Trials ID	Administration	Reference
Poly (lactic‐co‐glycolic acid)‐b‐poly(L‐lysine) Scaffold	Thoracic spinal cord injuries	Not yet started	US	NCT03762655	Interventional (Neuro‐Spinal Scaffold)	www.ClinicalTrials.gov
Bilaminar chitosan scaffold	CSF leakage	Not Applicable	Mexico	NCT03280849	Interventional	www.ClinicalTrials.gov
RGTA® (OTR4132)	Acute Ischemic Stroke	Not Applicable	France	NCT04083001	It is administered through intra‐arterial injection, in a one‐shot dose and most of the product is eliminated within 24 h	www.ClinicalTrials.gov
Ferumoxytol	Multiple sclerosis	Phase 1	US	NCT02511028	Ferumoxytol 510 mg (17 mL) IV	www.ClinicalTrials.gov
Self‐assembling peptide nanofiber hydrogels	Spinal cord injury	Not Applicable	China	NCT05967325	Stromal Vascular Fraction combined with Functional self‐assembling peptide nanofiber hydrogels	www.ClinicalTrials.gov

## 7 List of FDA‐Approved Commercially Available Therapies for Nerve Regeneration

Nerve conduits, wraps, and cuffs that have received FDA or CE approval are biodegradable.^[^ ^134^ ^]^ Nerve conduits made up of collagen, such as Neuroflex, NeuroMatrix, NeuroGen, NeuroWrap, NeuroMend, and RevolNerv, have been approved by the FDA.^[^ ^112^ ^]^ The survival rate of cells transplanted into hemi‐Parkinson's rats increased after the use of Cytodex, a collagen‐coated with dextran. Furthermore, Cytodex was applied without the requirement for immune suppression, maintaining the function of the transplanted cells.^[^ ^135^ ^]^ Currently, SaluTunnel and Salubridge are both made up of PVA and marketed by Atlanta, Georgia‐based SaluMedica L.L.C., and are the only nonbiodegradable scaffolds for nerves that are approved by the FDA. Both Neurolac and Neurotube are based on synthetic polyesters. They deteriorate between three and four years.^[^ ^134^ ^]^

## 8 Biological Response of the Biomaterial after Implantation at the Damaged Site

The implanted substances always cause a foreign body response, which is characterized by inflammation; over months or weeks, this response is followed by fibrotic encapsulation. Both acute inflammation and persistent fibrosis pose serious threats to the integrity and therapeutic effectiveness of implanted materials, which are intended to be used for treatment.^[^ ^136^ ^]^] Plasma proteins are first adsorbed on the surface of the implant nonspecifically, leading to an inflammatory reaction and subsequent healing of the wound. This process involves the release of proinflammatory mediators and the recruitment of leukocytes by immune cells, including neutrophils and macrophages. A fibrotic capsule surrounds the implant and collagen deposits because of the chronic inflammation that follows. The inflammatory response is typified by the presence of monocytes, lymphocytes, and fibroblasts. This capsule may impede tissue integration, nutrient exchange, and implant function, ultimately resulting in implant failure. For implant‐based treatments and tissue engineering devices, foreign body reactions (FBRs) pose serious obstacles that frequently call for further interventions or redesigns to be addressed.^[^ ^137^ ^]^

Over the past few decades, researchers have investigated a variety of ways to control immune responses toward implants due toFBRs. This is an enormous hurdle that impedes the development of implantable biomaterials and biomedical devices. Current approaches include altering the physical properties of the implanted biomaterials, adding biological agents or anti‐inflammatory medications to reduce inflammation, and changing the surface of the implanted biomaterials to contain bioactive components.^[^ ^137^ ^]^ Enhanced treatment efficacy may be achieved by combining certain properties, such as biodegradability, niche habitat formation, and adaptable size and shape attributes, and tailoring implanted materials to prioritize biocompatibility.^[^ ^138^ ^]^

During the developmental process, neurons are driven by various signaling pathways to stretch the axons toward the targeted site. Following trauma, neurons return to a state known as the “elongation mode”, which is akin to the ability of the PNS to regenerate itself. During injury, the influx of calcium ions quickly signals the development of new axons and changes in the calcium level impact the various downstream pathways that are necessary for regeneration. The process of regeneration is affected by changes in the ion concentrations because they produce cellular bioelectricity.^[^ ^139^ ^]^

Glial scarring and lesion cavities emerge because of both immediate and delayed cell death caused by brain injury. By introducing a scaffold into the injured site, the surrounding brain tissue is supported and provided with a platform for growth, regeneration of damaged tissue, neurite production of cells, and infiltration of cells. These scaffolds can also be designed to facilitate the transplantation of cells to replace missing circuitry or the controlled release of drugs for localized molecular delivery. Cells are added to 3D scaffolds that are intended to resemble the ECM of the native tissue of the damaged site, which regulates tissue shape and cell activity while promoting the flow of nutrients and growth factors. Microstructural changes in the scaffold significantly affect cortical reorganization, and greater cellular infiltration and alignment are facilitated when the scaffold pores face the host tissue. Cell adhesion, migration, and differentiation are improved when peptides such as RGD or PHSRNG6RGD are added to the surface of biomaterial scaffolds to resemble ECM components. When laminins are added to scaffolds, their cell‐binding domains, such as those of IKVAV and YIGSR, increase neurite extension and stimulate spinal cord regeneration. By architecturally imitating ECM and tubular structures, nanoscale scaffolds such as nanotubes and nanofibers support tissue bridging and axonal healing. Moreover, in animal models of brain damage, scaffolds in conjunction with NSCs are promising for brain repair because they improve cell survival, tissue regeneration, and functional recovery.^[^ ^138^ ^]^

## 9 Conclusion and Future Perspective

Biomaterials have great promise for neurological disease diagnosis and treatment since they are perfect at bridging the blood‒brain barrier. The primary advantages of biomaterials are their exceptional biocompatibility and elimination of the possibility of toxicity, which synthetic nanomaterials lack. Furthermore, because of their mechanical resilience, hydrogels are appealing scaffolds for tissue regeneration. The formulations for *in situ* gelling are perfect substitutes for invasive implantation procedures, which would normally call for a scaffold specifically tailored to accommodate uneven lesion cavities. The use of different biomaterials for various neurological disorders has shown promising outcomes in preclinical studies, but further investigation and optimization are needed for clinical settings. Despite the biomaterials being evaluated through both in vitro and in vivo studies, further research needs to be done in higher‐level animal models to evaluate their clinical potential before these materials can be used for human nerve regeneration. While these biomaterials offer various positive benefits, there are safety concerns regarding their use in humans, such as these biomaterials potentially causing oxidative stress or inflammation. Significant developments are anticipated soon in the field of biomaterials, which will enhance the treatment of neurological conditions in humans. The interactions between cells and materials can be enhanced by surface modification and enhanced nanofabrication processes. The future of biomaterials in neurological disorders holds exciting prospects, driven by advancements in precision medicine, tissue engineering, and bioelectronics. The convergence of these fields is set to the user into a new era of targeted, personalized, and effective treatments, offering hope for improved outcomes and enhanced quality of life for individuals affected by neurological disorders. The use of stem cells in combination with functional biomaterials has the potential to improve the treatment of neurological disorders. This integration may improve stem cell transplantation and differentiation processes. In the era of material design, new biodegradable biomaterials might result in the development of innovative therapeutic strategies for neurological disorders.

## Conflict of Interest

The authors declare no conflict of interest.
